# Crossed Corticospinal Facilitation Between Arm and Trunk Muscles Correlates With Trunk Control After Spinal Cord Injury

**DOI:** 10.3389/fnhum.2020.583579

**Published:** 2020-10-23

**Authors:** Shin-Yi Chiou, Paul H. Strutton

**Affiliations:** ^1^Sport, Exercise, and Rehabilitation Sciences, College of Life and Environmental Sciences, University of Birmingham, Birmingham, United Kingdom; ^2^The Nick Davey Laboratory, Division of Surgery, Department of Surgery and Cancer, Faculty of Medicine, Imperial College London, London, United Kingdom

**Keywords:** trunk control, functional reaching, spinal cord injury, transcranial magnetic stimulation, electromyography, erector spinae

## Abstract

**Objective**: To investigate whether crossed corticospinal facilitation between arm and trunk muscles is preserved following spinal cord injury (SCI) and to elucidate these neural interactions for postural control during functional arm movements.

**Methods**: Using transcranial magnetic stimulation (TMS) in 22 subjects with incomplete SCI motor evoked potentials (MEPs) in the erector spinae (ES) muscle were examined when the contralateral arm was at rest or performed 20% of maximal voluntary contraction (MVC) of biceps brachii (BB) or triceps brachii (TB). Trunk function was assessed with rapid shoulder flexion and forward-reaching tasks.

**Results**: MEP amplitudes in ES were increased during elbow flexion in some subjects and this facilitatory effect was more prominent in subjects with thoracic SCI than in the subjects with cervical SCI. Those who showed the increased MEPs during elbow flexion had faster reaction times and quicker anticipatory postural adjustments of the trunk in the rapid shoulder flexion task. The onset of EMG activity in ES during the rapid shoulder flexion task correlated with the trunk excursion in forward-reaching.

**Conclusions**: Our findings demonstrate that crossed corticospinal facilitation in the trunk muscles can be preserved after SCI and is reflected in trunk control during functional arm movements.

## Introduction

Impaired voluntary control of trunk muscles is commonly seen following human spinal cord injury (SCI; Potten et al., 1999; Milosevic et al., 2015) and this can compromise activities that involve interactions between limb and trunk muscles (Kukke and Triolo, 2004; Desroches et al., 2013). For example, reaching for, or catching an object, requires the activity of trunk muscles before or concurrent with voluntary arm movements (Kaminski et al., 1995; Aruin et al., 2015). In locomotion, trunk muscles are involved in maintaining the center of mass within the base of support of the body and hence keep the body stable (Nataraj et al., 2012; Moraud et al., 2018). Evidence has shown neural interactions between the pathways projecting to the arm and trunk muscles. Human studies using non-invasive transcranial magnetic stimulation (TMS) over the primary motor cortex (M1) demonstrated increased amplitudes of motor evoked potentials (MEPs) of the trunk muscles before or during the arm movements (Davey et al., 2002; Chiou et al., 2016, 2018a). Further, we have recently shown that this crossed corticospinal facilitation between trunk extensor and proximal arm muscles is mediated, in part, cortically (Chiou et al., 2018b). Also, the facilitatory effect was more prominent during elbow flexion (Chiou et al., 2018b), highlighting that the neural interactions between the arm and trunk muscles can be influenced by the task. These findings suggest that crossed facilitation may underpin functionally relevant arm-trunk interactions.

Damage to the spinal cord often results in the degeneration of corticospinal axons (Hill et al., 2001; Hassannejad et al., 2019) which can alter characteristics of physiological responses of the corticospinal tract (Ellaway et al., 2007; Oudega and Perez, 2012). For instance, it has been demonstrated that in people with SCI amplitudes of MEPs are reduced, latencies are prolonged, and motor thresholds are increased compared to uninjured controls (Davey et al., 1998; Ellaway et al., 2007). Alterations in corticospinal function have been shown to relate to the functional recovery of limbs after SCI (Belci et al., 2004; Wirth et al., 2008; Sangari et al., 2019). Previous work on the trunk has shown that the MEP latencies in paraspinal muscles were prolonged in SCI patients with severe motor impairment compared with those with mild motor impairment (Ogura et al., 2003). Crossed facilitation between the limbs is altered in people with SCI, and the effect varied with the level of injury (Bunday and Perez, 2012; Bunday et al., 2013). However, how the crossed corticospinal facilitation between the arm and trunk muscles is affected by SCI and whether this is related to the function of arm-trunk movements remain unknown. We hypothesized that crossed corticospinal facilitation between arm and trunk muscles will be reduced in subjects with SCI due to the damage to the spinal cord, and the extent of crossed corticospinal facilitation of the trunk muscles during arm contractions would correlate with trunk motor function during functional arm movements.

To test our hypotheses, we examined the amplitudes of MEPs in trunk extensor muscles elicited by TMS over M1 with the arm muscles at rest and during voluntary contractions. We further investigated the influence of this crossed corticospinal facilitation on the trunk during functional arm movements such as reaching.

## Materials and Methods

### Subjects

Twenty-two subjects with incomplete SCI [mean age ± standard deviation (SD): 51.4 ± 18.1 years; range: 21–82 years; 13 male] participated in the study. All subjects gave written informed consent before participation and the study was approved by West Midlands—South Birmingham Research Ethics Committee. The study was performed following the Declaration of Helsinki. Individuals with SCI had a chronic (≥1 year), cervical (C2–6), or thoracic (T3–10) injury. Eleven individuals were categorized by the American Spinal Injury Association Impairment Scale (AIS) as AIS C; the other 11 individuals as AIS D. Trunk function of all subjects was assessed using the trunk impairment scale (TIS; Verheyden et al., 2004), with the score ranging from 0 (severe impairment) to 23 (no impairment; 13.2 ± 6.9). Hand dexterity of the SCI subjects was assessed using the nine-hole peg test (Belci et al., 2004). The time required to complete the test was longer in the more affected hand/non-dominant hand than in the less affected hand/dominant hand in all subjects (cervical SCI: less affected vs. more affected: 55.65 ± 34.90 vs. 75.84 ± 46.64 s *t* = −3.45, *p* = 0.009; thoracic SCI: 20.69 ± 3.76 vs. 22.16 ± 3.65 s *t* = −4.58, *p* = 0.002). Time differences between hands to complete the task were greater in cervical SCI (20.18 ± 17.53 s than in thoracic SCI (1.47 ± 0.96 s *t* = 3.20; *p* = 0.013). The demographic data of all participants are listed in Table 1. The data from sixteen healthy subjects (eight male; 29.7 ± 10.9 years; range: 21–52 years) collected previously (Chiou et al., 2018b) are included in this study for comparison to that from subjects with SCI.

**Table 1:** Demographic data of participants.

Participants	Age, year	Gender	AIS	Level	Etiology	Time since	TIS	NHPT	NHPT
						the injury, year		(less affected/D; s)	(affected/ND; s)
P1	40	M	D	C3/4	T	4	17	112.5	120.72
P2	58	M	C	T4	T	6	2	26.01	27.89
P3	21	M	D	C4	T	1	18	33.35	N/A
P4	54	M	D	C4/5	T	1	23	19.53	24.90
P5	54	F	C	T3/4	NT	4	5	17.53	18.81
P6	31	M	C	T7	NT	16	15	24.76	25.74
P7	69	F	D	C4	T	5	12	38.20	N/A
P8	26	F	C	C5/6	T	11	13	25.05	N/A
P9	37	M	C	C5/6	T	20	4	106.74	154.12
P10	32	F	C	T7	T	1.2	13	18.93	19.63
P11	33	F	C	C5/6	T	5	4	73.39	114.12
P12	64	F	C	T3	T	3	13	16.09	18.9
P13	73	M	D	C1/2	T	1	19	51.82	90.8
P14	41	F	D	C4	T	13	15	50.37	70.14
P15	65	F	D	C1/2	NT	2	21	24.90	26.20
P16	56	M	D	T10	T	5	23	25.23	25.67
P17	82	M	D	C4/5	T	2	12	36.46	43.73
P18	42	M	C	T10	T	32	13	17.19	17.92
P19	62	M	C	T3	T	1	5	20.09	21.42
P20	69	M	D	T4	T	1	21	20.26	23.46
P21	55	M	C	C3/4	T	3	2	209	N/A
P22	60	F	D	C1/2	T	1	21	25.22	37.8

*AIS, American Spinal Injury Association Impairment Scale; TIS, Trunk impairment Scale; T, traumatic spinal cord injury; NT, non-traumatic spinal cord injury; M, male; F, female. NHPT, night-hole peg test; D, dominant; ND, non-dominant; N/A, unable to carry out the test*.

### EMG Recordings

EMG was recorded bilaterally from erector spinae (ES) at the 12^th^ thoracic vertebral level (T12) and anterior deltoid (AD), and unilaterally from the biceps (BB) and triceps (TB) brachii of the less affected arm or the dominant arm through pairs of surface electrodes (Ag-AgCl; 10 mm diameter, CareFusion, UK) secured to the skin over the belly of each muscle in line with the orientation of the muscle fibers. Electrodes were positioned 3 cm lateral to the spinous processes with an inter-electrode distance of 2 cm. To determine the less affected arm, manual muscle testing (MMT) was performed on each participant to examine the strength of elbow flexors and elbow extensors by an experienced physiotherapist (the author S.Y. Chiou). The side with greater muscle strength was defined as the less affected arm. If the muscle strength between sides was the same based on the MMT, the side participants reported as being less affected or the dominant side was used. The signals were amplified (×1,000), filtered (10–1,000 Hz), and sampled at 2 kHz for off-line analysis (CED Power1401 with Signal software, Cambridge Electronic Design, Cambridge, UK).

### Experimental Setup

**Figure 1:**
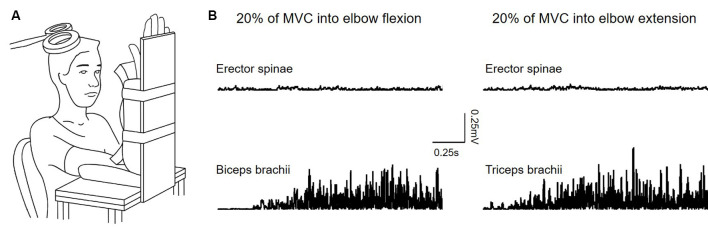
**(A)** Experimental setup. **(B)** Raw rectified electromyographic (EMG) activity from each of the muscles tested during 20% of maximal voluntary contraction (MVC) into elbow flexion (recording from the biceps brachii) and elbow extension (recording from the triceps brachii).

Subjects were seated in an armchair with the shoulder and elbow of the less affected arm/dominant arm flexed to 90° and the forearm supinated. A custom-made arm device was used to maintain the position of the arm (Figure 1A). At the beginning of the experiment, all subjects performed three brief isometric maximal voluntary contractions (MVCs) for 3–5 s into elbow flexion and extension, separated by 30 s of rest. MVCs were quantified using EMG activity in the biceps brachii (BB) and triceps brachii (TB) muscle during elbow flexion and elbow extension, respectively. During MVCs subjects received constant verbal encouragement to ensure maximal performance. MVCs for the ES were collected in a prone position with the pelvis and legs of the subjects secured by the researchers. The level of EMG calculated from averaging the highest mean rectified EMG activity in 0.5 s during the MVCs of each muscle was defined as the maximal voluntary contraction (MVC) of that muscle. Testing was performed when the less affected arm remained at rest (defined as the rest condition) or when performing 20% of MVC into elbow flexion and extension. Background EMG in the ES during the arm contractions was measured and matched during the rest condition (Figure 1B). Rectified EMG activity from the ES and arm muscles was displayed continuously on a computer screen, with feedback provided verbally to ensure that participants produced consistent levels of background EMG activity during neurophysiological measurements. Familiarization trials were given to ensure that all subjects were able to perform the tasks using the required level of EMG activity. If subjects reported increased muscle tone or reflexes (i.e., clonus) in muscles of limbs and/or the trunk during the testing, breaks were given and the position of the body was re-adjusted to eliminate the influence of the muscle tone and reflexes on the data.

To quantify the function of trunk muscles, all subjects undertook a rapid shoulder flexion task and a forward reaching task in a seated position without the support of their torso. For the rapid shoulder flexion task, subjects were instructed to raise both arms to 90° as fast as possible in response to a visual cue 10 times (Hodges et al., 1999). For the forward reaching task, subjects sat on a custom made chair with an embedded force plate (Kistler Type 9286B, Kistler Instrumente AG, Winterthur, Switzerland) and had reflective markers attached over the ulnar styloid process of the less affected arm and spinal process of the first thoracic vertebra (Field-Fote and Ray, 2010). Their hips and knees were in 90° flexion and feet were placed flat on a step. Subjects were instructed to reach forward as far as possible at their comfortable speed using the less affected arm three times. A 10 camera 3-D motion capture system (Vicon, Oxford Metrics, Oxford, UK) operating at 100 Hz was used to capture the positions of the reflective markers. Force plate data were recorded using an analog signal data acquisition card provided with the Vicon system and the Vicon Nexus software at a sampling rate of 1,000 Hz and synchronized with the motion data.

### Transcranial Magnetic Stimulation

TMS was delivered *via* a Magstim 200^2^ monophasic stimulator (The Magstim Company Ltd., UK) through a figure-of-eight coil (loop diameter 70 mm), handle pointing backward and 45 (degrees) away from the midline (Ferbert et al., 1992; Chiou et al., 2018b). The ES muscle contralateral to the less affected arm was chosen as the recording site as functionally activating the upper limb of one side of the body results in increased activation of the contralateral ES (Davey et al., 2002). The optimal position for eliciting an MEP in the ES muscle (hot spot) was determined by moving the coil in small steps in the area corresponding to the M1. The hot spot was defined as the area in which the largest MEP in the ES was evoked with the lowest intensity (Rothwell et al., 1999). Once the hotspot was found and confirmed by several stimuli as a consistency check, the position of the coil was marked on the scalp to ensure consistent placement of the coil throughout the experiments. Since our subjects with SCI had an injury above the level of T12, the recording ES muscle was below the level of injury in all the participants.

### Motor Evoked Potentials

The active motor threshold (AMT) of the ES muscle was established while subjects were seated without the support of their trunk. The threshold was defined as the lowest intensity of TMS that evoked visible MEPs in at least three of six consecutive trials. Due to injuries to the spinal cord, the majority of subjects (*n* = 20) had an AMT above 85% maximal stimulator output (MSO) and thereby required intensity of 100% MSO for the testing (Ellaway et al., 2007). There were only two SCI participants who had AMT less than 85%. Therefore, the intensity of 120% AMT was used for testing in these two participants to ensure clear and consistent recordings in the ES. The range of intensities used for all SCI participants was 78–100% MSO. AMT of the ES muscle was 74.92 ± 12.52% MSO in the controls. TMS pulses were delivered at 4 s intervals in sets of 10. Breaks were given if needed. Ten MEPs were tested during each voluntary contraction and the rest condition.

### Data Analysis

Peak-to-peak MEP amplitudes from ES were averaged for each condition and expressed as a percentage of the control ES MEP amplitude (when the upper limb was at rest). The presence of crossed facilitation was defined as the averaged MEP size which was greater during voluntary contractions of the arm (i.e., >100%) than during the rest condition. Conversely, an averaged MEP size less than 100% during the task concerning the rest condition was defined as the absence of crossed facilitation. EMG obtained from ES, BB, and TB during MVCs was rectified and calculated as mean EMG in a 500-ms window. Pre-stimulus ES EMG was rectified and calculated as mean EMG in a 100-ms window before the stimulus and presented as a percentage of MVC. The time of onset of EMG activity in AD and ES during the shoulder flexion task was calculated as the time at which mean rectified EMG activity increased over 3 SDs above the mean pre-stimulus EMG level in a 50-ms window (Hodges et al., 1999). The reaching distance was measured from the maximal displacement of the wrist marker on the y-axis (anterior-posterior direction; Field-Fote and Ray, 2010). Trunk movement in reaching was measured from the maximal displacement of the trunk marker (Field-Fote and Ray, 2010). The maximal displacement of the center of pressure (CoP) in anterior-posterior and in medial-lateral directions during forward-reaching was calculated (Lemay et al., 2014).

### Statistical Analysis

Data were analyzed using SPSS version 24 (IBM Corp., Armonk, NY, USA). The Shapiro-Wilk test was used to test whether the data were normally distributed; the Mauchly test was used to test sphericity. When the data were not normally distributed (*p* < 0.05), non-parametric tests were applied; Mann–Whitney *U* tests and Wilcoxon signed-rank tests were used for between-group and within-group comparisons, respectively. When sphericity could not be assumed the Greenhouse–Geisser correction statistic was used. Repeated-measures analysis of variance (ANOVA) was performed to determine the effect of condition (rest, elbow flexion, and elbow extension) on MEP size and mean rectified EMG in the ES muscle. A mixed-model repeated-measures ANOVA was employed to examine the effect of condition and the interaction between condition and group (subjects with SCI vs. controls). *Post hoc* tests with Bonferroni’s correction were applied for significant comparisons. Independent *t-tests* were used to examine differences in onsets of EMG activity in AD and ES during the shoulder flexion task, reaching distance, and trunk movement in reaching between subjects with and without increased ES MEPs during the arm contractions. Regression analyses were employed to identify the relationship between the crossed facilitation in the ES (continuous data) and level of injury (categorical data). Correlation analyses were employed to identify the relationship between the size of ES MEP during elbow flexion and trunk function (i.e., onset of EMG activity in ES during the shoulder flexion task). Significance was set at *p* < 0.05. Group data are presented as the mean ± SD in the text.

## Results

### EMG

Results demonstrated that mean rectified EMG of the ES during MVCs was lower in the SCI participants (recording site: 0.10 ± 0.07 mV; contralateral side: 0.09 ± 0.05 mV) than in the controls (left ES: 0.33 ± 0.14 mV; right ES: 0.33 ± 0.17 mV; both *p* < 0.001). This indicates impairment in motor function of bilateral ES in subjects with SCI.

Repeated measures ANOVA showed no effect of condition (*F*_(2,40)_ = 0.09; *p* = 0.92) on mean rectified EMG activity in the contralateral ES muscle (rest: 16.85 ± 13.03% MVC; elbow flexion: 16.38 ± 10.70%; elbow extension: 17.40 ± 16.11%). This result demonstrated that mean rectified ES EMG activity was constant when the arm was at rest or performed 20% of MVC into elbow flexion and elbow extension, in agreement with our previous work in healthy subjects (Chiou et al., 2018b). We also found no difference in the level of muscle contraction exerted by BB and TB between elbow flexion (18.72 ± 9.53% BB MVC) and elbow extension (17.58 ± 9.33% TB MVC; *p* > 0.05).

### MEPs

**Figure 2:**
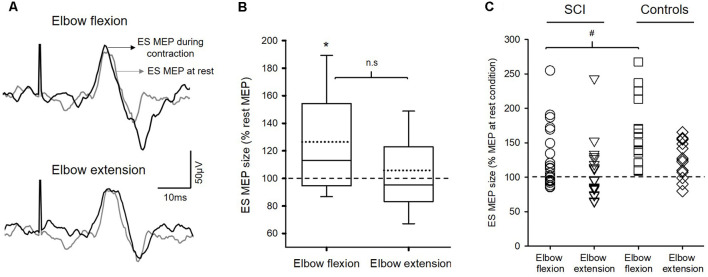
Motor evoked potentials (MEPs). **(A)** Raw traces recorded from erector spinae muscles (ES) of a representative subject with T4 incomplete spinal cord injury (SCI). Traces show the average of 10 MEPs in the ES muscle at rest (gray traces) and during 20% of MVC of arm contractions (black traces). **(B)** Group data (*n* = 22) showing MEPs in ES between conditions. Solid lines indicate median values; dotted lines indicate mean values. The box is interquartile range; error bars denote maximum and minimum values. The horizontal dashed line represents the size of the ES MEP in the rest condition. Note that the amplitudes of MEP in the ES muscle increased during elbow flexion but not during elbow extension. *p < 0.05, comparison between rest and the voluntary contractions. n.s, nonsignificant. **(C)** Note that SCI participants (circle and reversed triangle) who show increased MEPs during either elbow flexion or extension have the amount of facilitation similar to the controls (square and diamond). Also, the majority of SCI participants show increases in ES MEPs during elbow flexion compared with rest. ^#^*p* < 0.05, comparison between SCI subjects and the controls.

Figure 2A illustrates traces of averaged MEPs elicited by TMS over the M1 in the ES muscle from a representative subject. Note that the size of MEP in the ES muscle was increased during elbow flexion but not during elbow extension compared to the rest.

Repeated-measures ANOVA revealed an effect of condition (*F*_(2,40)_ = 4.91; *p* = 0.012) on ES MEP size in subjects with SCI (*n* = 22; Figure 2B). Note that more participants showed increased ES MEP size during elbow flexion than during elbow extension (Figure 2C). *Post hoc* tests showed that ES MEP amplitude increased during elbow flexion (126.49 ± 43.63% MEP at rest; 0.13 ± 0.11 mV; corrected *p* = 0.03) but not elbow extension (105.80 ± 39.65%; 0.11 ± 0.08 mV; *p* = 0.51) compared with the rest condition (0.11 ± 0.07 mV). There was no difference in ES MEP amplitude between elbow flexion and elbow extension (corrected *p* = 0.093). Since some SCI subjects did not show a facilitatory effect on the ES (i.e., MEP size <100% during the task concerning the rest condition), a subgroup analysis was performed on the subjects (*n* = 14) who show increased MEPs in ES during either elbow flexion or elbow extension. Results revealed the same effect as the main findings; ES MEPs were increased during elbow flexion compared with rest (*p* = 0.002) but not different from that during elbow extension (*p* = 0.12). Additionally, the same results were found in the subgroup of patients with thoracic SCI; increased ES MEPs during elbow flexion (*p* = 0.02) but no difference during elbow extension (*p* = 0.35) compared with rest or between elbow flexion and extension (*p* = 0.24). There was no difference in rectified EMG of BB or TB during MVC between subjects with thoracic SCI and the controls (BB MVC: *Z* = −0.28; *p* = 0.80; TB MVC: *Z* = −1.61; *p* = 0.12), confirming that motor function of BB and TB was intact in our cohort with thoracic SCI.

**Figure 3:**
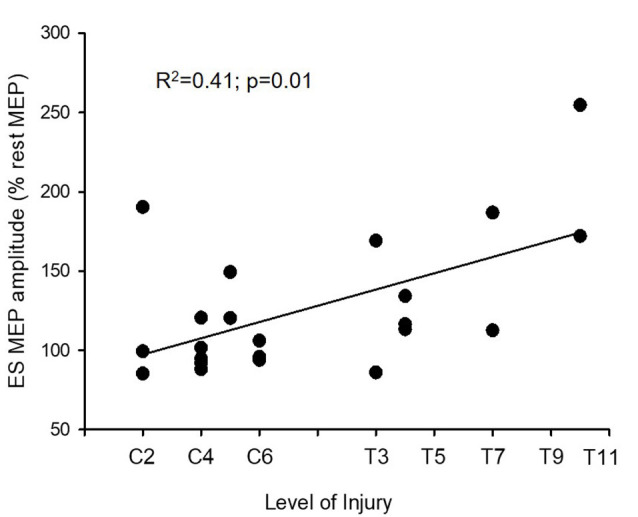
Motor evoked potentials (MEPs) and level of injury. The level of injury correlates with amplitudes of MEP in the erector spinae (ES) muscle during elbow flexion (*n* = 22). The ordinate shows the size of the ES MEP during the elbow flexion (as a % of the ES MEP obtained at rest). Note that the crossed facilitatory effect of the arm contraction on the trunk muscle is greater in subjects with a more caudal injury, near the recording muscle (the ES muscle at the 12^th^ thoracic vertebral level, T12).

We compared the amount of facilitation in ES MEPs between the previous control data and the SCI subjects. A mixed-model repeated measures ANOVA revealed an effect of condition (*F*_(2,70)_ = 23.19; *p* < 0.001) and an interaction between group and condition (*F*_(2,70)_ = 3.71; *p* = 0.03) on the size of ES MEP. *Post hoc* test showed that the amplitudes of ES MEP during elbow flexion were smaller in SCI subjects (126.49 ± 43.63%) than in the controls (164.31 ± 48.58%; Figure 2C). However, the amplitude of ES MEP during elbow extension did not differ between SCI subjects and the controls. Regression analysis revealed that changes in ES MEPs during elbow flexion correlated with the level of injury (*R*^2^ = 0.41; *p* = 0.01; Figure 3); subjects with higher levels of injury showed less crossed corticospinal facilitation during elbow flexion. To further examine whether the larger facilitatory effect was due to less impairment in arm muscles, we first examined the relationship between the raw amplitude of ES MEPs during elbow flexion and the amplitude of rectified EMG during BB MVCs in subjects with cervical SCI. BB MVCs were lower in cervical SCI than in the controls (*Z* = −2.28; *p* = 0.02). There was no correlation between the amplitude of ES MEP and BB MVCs (*p* = 0.31). We further performed a subgroup analysis in subjects with thoracic injuries (i.e., those in which BB is not affected by the injury). This was confirmed by the statistical analysis as BB MVCs were not different between thoracic SCI and the controls (*Z* = −0.28; *p* = 0.80). Descriptive statistics showed that amplitudes of ES MEP (% of MEP at rest) during elbow flexion tasks were 119.50 ± 30.38%, 160.54 ± 37.21%, and 213.42 ± 58.41% in subjects with an injury at T4 (*n* = 5), at T7 (*n* = 2), and T10 (*n* = 2), respectively. Note that the size of ES MEP is greater in those with T11 injury than in those with injuries at T4. Regression analysis yielded a trend of correlation between changes in ES MEPs during elbow flexion and the level of injury similar to the main findings (*R*^2^ = 0.47; *p* = 0.06) due to the smaller sample size and unequal numbers across categories. Our findings suggest that the level of injury may affect the magnitude of crossed facilitation after SCI.

### Trunk Function

To investigate the functional relevance of the crossed facilitation in the ES during elbow flexion, subgroup comparisons between SCI subjects with (*n* = 14) and without (*n* = 8) crossed facilitation were performed on measurements of trunk function. Notably, seven subjects of 13 with cervical SCI showed no crossed facilitation, where only 1 subject of 9 with thoracic SCI showed no increase in ES MEPs during elbow flexion.

**Figure 4:**
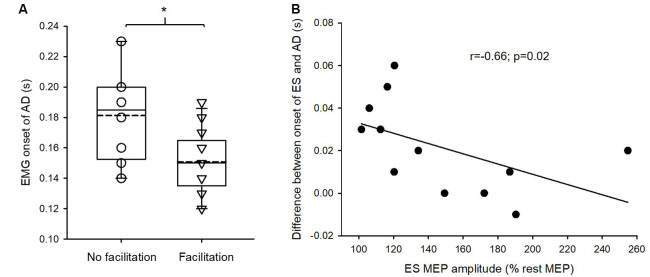
Electromyography (EMG) of anterior deltoid (AD) and erector spinae (ES) in the rapid shoulder flexion task. **(A)** The onset of EMG activity in AD is earlier in patients with crossed facilitation (*n* = 14) than in those without (*n* = 8), indicating that patients with the crossed facilitation react quicker to a visual cue. Solid lines indicate median values; dotted lines indicate mean values. The box is interquartile range; error bars denote maximum and minimum values. **(B)** Increased the size of motor evoked potential (MEP) in the ES muscle correlates with the onset of EMG activity in ES concerning AD during the rapid shoulder flexion task in patients with crossed facilitation. This indicates that patients who have preserved crossed facilitation of the trunk muscles show the better function of anticipatory postural adjustments during functional arm movements. *p < 0.05 in comparison between subgroups.

Mann–Whitney *U* test revealed that the onset of EMG activity in AD during the rapid shoulder flexion task was significantly earlier in SCI subjects with crossed facilitation than in those without crossed facilitation (*Z* = −2.32; *p* = 0.02; Figure 4A). This suggests that SCI subjects with increased ES MEPs during elbow flexion reacted faster to the visual cue compared with those without the facilitation. Additionally, in those with crossed facilitation, the onset of EMG activity in ES concerning AD during the rapid shoulder flexion task correlated with the increased ES MEP size during elbow flexion (*ρ* = −0.66; *p* = 0.02; Figure 4B). This indicates that subjects with SCI having greater crossed facilitation in the ES had quicker anticipatory postural adjustments of the trunk during the functional arm movements.

**Figure 5:**
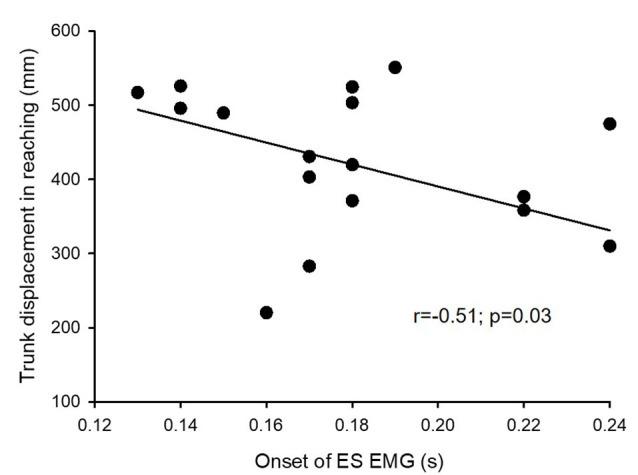
Electromyography (EMG) and trunk trajectory. Onset of EMG activity in the erector spinae (ES) muscle during the rapid shoulder flexion task correlates with a maximum displacement of trunk trajectory in reaching. This indicates that patients who have better anticipatory postural adjustments of the trunk have a greater reaching distance.

Further, the onset of EMG activity in ES during the rapid shoulder flexion task correlated with a maximal displacement of trunk movement in forward-reaching (*r* = −0.51; *p* = 0.03; Figure 5). This indicates that subjects with SCI who had quicker anticipatory postural adjustments of the trunk during the functional arm movements were able to move their trunk further in forward-reaching. We did not find a correlation between the magnitude of ES MEP during elbow flexion and forward-reaching distance (*r* = 0.22; *p* = 0.34) or the trunk impairment scale (*r* = 0.27; *p* = 0.22) in SCI participants.

## Discussion

We demonstrate that corticospinal excitability of a trunk muscle was increased during voluntary activation of elbow flexors in subjects with SCI and the magnitude of this facilitatory effect correlated with the level of injury, with greater facilitation observed in subjects with thoracic SCI compared with subjects with cervical SCI. The same effect was not observed during voluntary activation of elbow extensors, however. Furthermore, subjects who showed increased corticospinal excitability of the trunk muscles had faster reaction time and quicker anticipatory postural adjustments of the trunk during the functional arm movement compared to those who did not. Also, subjects with quicker anticipatory postural adjustments of the trunk moved their trunk further forward in the reaching task. Our findings provide the first evidence, to the best of our knowledge, that neural interactions between arm and trunk muscles influence control of the trunk during functional arm movements following SCI.

### Crossed Facilitation of the Trunk Muscle Correlates With the Function of the Trunk After SCI

Crossed corticospinal facilitation has been widely observed in healthy humans (Hortobágyi et al., 2003; Perez and Cohen, 2008; Chiou et al., 2018b). It is suggested that this facilitatory effect is related to interlimb coordination and motor performance (Carson et al., 2008; Lee et al., 2010). Damage to the corticospinal tract can interrupt motor function and alter the neurophysiology of the central nervous system. A previous study in human cervical SCI showed that crossed facilitation in a small hand muscle is absent during voluntary contractions of the contralateral hand muscle even though subjects could voluntarily move the hands (Bunday and Perez, 2012). Their findings suggest that crossed facilitation may not relate to motor status after SCI. While all our participants were able to maintain an unsupported seated position and had activation of their trunk muscles, we found that subjects who showed increased corticospinal excitability of the trunk muscles during the elbow flexion could move their arms faster and activate the trunk muscles earlier concerning their arms in response to a visual cue. It is well documented that trunk muscles are activated concurrently or before a fast arm movement to maintain postural stability, this activation is termed anticipatory postural adjustments (Hodges et al., 1999; Chiou et al., 2018a). Delays in trunk muscle activation often affect the use of the arms (Bruttini et al., 2016; Collins et al., 2018). Hence, our results indicate that subjects with greater crossed facilitation of the trunk muscles had a quicker reaction time of the arms and better anticipatory postural adjustments of the trunk during the functional arm movements. This supports the notion that crossed corticospinal facilitation of the trunk muscles is involved in functional coordination between arm and trunk movements in people with SCI.

### Altered Crossed Facilitation of the Trunk Muscles After SCI

We found that the facilitatory effect of contracting arm muscles on corticospinal excitability to the trunk muscle is reduced in subjects with SCI. We recently reported that arm contractions at 20% of MVCs can increase MEP size in the trunk muscles, and this facilitatory effect was more prominent during elbow flexion than during elbow extension in healthy subjects (Chiou et al., 2018b). In the subjects with SCI, the increase in ES MEPs was found during elbow flexion but not during elbow extension, and there was no difference in ES MEPs between elbow flexion and elbow extension, albeit there was a tendency of greater MEPs in ES during elbow flexion than during elbow extension. One explanation could be the extent of damage to descending pathways. Alterations in electrophysiological measures, such as delayed MEP latencies, increased MEP threshold, and reduced MEP amplitude after human SCI have been reported (Davey et al., 1999; Ellaway et al., 2007). Evidence has shown that electrophysiological changes after SCI are associated with the degeneration of damaged corticospinal axons. Demyelination of corticospinal axons is commonly observed in the injured spinal cord (Bunge et al., 1993), affecting large-diameter axons (Quencer et al., 1992) which are activated by TMS (Petersen et al., 2003, 2010). Other pathological changes including decreased number of myelinated corticospinal axons and retrograde degeneration of the injured corticospinal tract (Fishman, 1987; Yamamoto et al., 1989) also contribute to altered TMS findings. The reduced crossed corticospinal facilitation observed in our subjects could be related to a reduced number of corticospinal axons reaching the spinal motoneurons (Davey et al., 1999; Ellaway et al., 2007). Another physiological mechanism involved in modulating the crossed corticospinal facilitation could be the propriospinal interneurons projecting to spinal cord segments below the injured site (Pierrot-Deseilligny, 1996). Animal studies have shown that after an SCI propriospinal commissural interneurons can reconnect the injured corticospinal tract, forming new intraspinal circuits to receive descending commands from the corticospinal motor system (Fouad et al., 2001; Bareyre et al., 2004; Fenrich and Rose, 2009). Evidence suggests that this spontaneous axonal regeneration may contribute to functional recovery after incomplete SCI (Courtine et al., 2008). In agreement, we found that SCI subjects with crossed facilitation could voluntarily move their arms faster in response to a visual cue.

A key finding is the lack of muscle dependency of crossed facilitation in the subjects. Our results indicate that an SCI has differential influences on BB and TB interacting with corticospinal projections to the ES muscle. The impairment seems to be greater in the TB than in the BB. Following SCI neural reorganization occurs at both cortical and spinal levels (Oudega and Perez, 2012) which can alter neural control of elbow flexor and extensors, resulting in a lack of muscle dependency in the crossed facilitation. Although crossed facilitation induced by strong voluntary contractions involves both cortical and spinal mechanisms, it is predominantly mediated cortically during low levels of contractions (Stedman et al., 1998; Chiou et al., 2018b). One could argue that strong levels of voluntary activity are likely to induce a greater amount of crossed facilitation (Muellbacher et al., 2000; Perez and Cohen, 2008). Elbow extensors are often more impaired than elbow flexors after SCI. However, we did not observe crossed facilitation during elbow extension in subjects with thoracic SCI who had normal function of upper limbs, suggesting that the change is likely due to the neural reorganization in the CNS following the injury.

### Crossed Facilitation of a Trunk Muscle Correlates With the Level of Injury

A next important finding is a correlation between magnitudes of the crossed corticospinal facilitation in the ES and the level of injury. We demonstrate that subjects with an injury closer to the recording site (T12) showed greater ES MEPs during the elbow flexion than those with an injury further away from the recording site. Following SCI, Wallerian degeneration occurs at the injury site and extends caudally (Bresnahan, 1978; Hill et al., 2001). This can result in the reduced neural drive from the contralateral corticospinal tract, thereby reduced crossed corticospinal facilitation. Subjects with cervical SCI are likely to have greater axonal degeneration, proportionally, in the corticospinal tract which may explain reduced crossed corticospinal facilitation in the trunk muscle. Also, several lines of evidence from animal studies have shown different responses to the injury at the thoracic spinal cord and the cervical spinal cord (Nashmi and Fehlings, 2001; Lee et al., 2016), suggesting a relationship between the level of injury and the pathophysiology of SCI.

Our findings of crossed facilitation in the trunk muscles induced by the elbow flexors are in keeping with a previous study showing that the crossed facilitation could be present below the injury site (Bunday et al., 2013). However, we did not observe an aberrant increase in the crossed facilitation in the trunk muscles in our subjects, as shown in prior work that MEPs in the muscles distant from the injury site (>15 segments) were increased aberrantly (Bunday et al., 2013). A previous study investigated crossed facilitation between limb muscles; the contracting muscles and the recording muscles are at the same spinal level and can be both above, at, or below the injury site (Bunday et al., 2013). We investigated crossed facilitation between arm and trunk muscles, with the recording site always below the spinal level of the injury site and the contracting muscle either above, at, or below the injury. Also, the authors of the previous study (Bunday et al., 2013) observed increased *F*-wave amplitudes together with aberrantly increased MEPs at the muscles distant from the injury site (Bunday and Perez, 2012). This may indicate the involvement of spinal mechanisms as the contraction level in that study was 70% MVC. The contraction level used in our study was low and the contribution from the spinal cord is likely to be less, albeit there is still a possibility that different mechanisms are involved in subjects due to the reorganization of the spinal cord following SCI. One could argue that subjects with cervical SCI have impaired elbow flexors which may cause a smaller effect of crossed facilitation in the trunk muscle. However, this seems unlikely, given that we did not find a correlation between the amount of crossed facilitation during elbow flexion and levels of EMG during MVCs of BB in subjects with cervical SCI. Also, we found the same correlation in subjects with thoracic SCI who showed similar EMG levels of BB MVCs to that reported in healthy controls (Chiou et al., 2018b), indicating a minimum influence of the MVCs of the arm muscles on crossed facilitation of the trunk muscles. We, therefore, suggest that the impaired interaction between arm and trunk muscles observed in the SCI subjects reflects corticospinal reorganization after the injury.

### Clinical Applications

The presence of axonal degeneration after SCI is well established (Bunge et al., 1993; Buss et al., 2004). Demyelination and progressive atrophy of surviving corticospinal axons might contribute to impaired voluntary motor output after SCI. A key goal of rehabilitation, either *via* therapeutic exercise or stimulation over the motor cortex (Tazoe and Perez, 2015) or spike-timing-dependent plasticity protocols (Long et al., 2017), is to strengthen corticospinal transmissions in spared spinal pathways, thereby improving motor output (Thickbroom et al., 2006; Long et al., 2017). Findings from prior work suggest that crossed corticospinal facilitation could be applied to training for improved arm function in patients with neurological disorders (Kowalczewski et al., 2011; Hamzei et al., 2012). Impaired trunk control is often observed in patients with stroke (Verheyden et al., 2006) or spinal cord injury (Field-Fote and Ray, 2010). Our findings highlight the potential to use the arms for increased neural interactions between the arm and trunk muscles, leading to improved functional outcomes. This is substantiated by our results that those with less crossed facilitation between arm and trunk muscles showed poorer trunk function during reaching. As such, crossed facilitation between arm and trunk muscles presents an opportunity for trunk rehabilitation, albeit the extent to which these interactions may be exploited will likely depend on the level of injury. Whether the use of exercise targeting the upper limbs can improve the function of the trunk in subjects with SCI remains to be tested.

## Conclusions

We demonstrate for the first time that crossed corticospinal facilitation between arm and trunk muscles is present in some subjects with SCI, with a stronger facilitatory effect in subjects with thoracic SCI. Subjects with crossed facilitation in the trunk muscle show a quicker reaction when raising the arms in response to the visual cue, indicating crossed facilitation between arm and trunk being used for functional gain following SCI. This supports the notion that the crossed corticospinal facilitation in the trunk muscle during voluntary contractions of the arm is relevant to limb and trunk interactions during upper limb movements.

## Data Availability Statement

The datasets presented in this article are not readily available because patient data need to be handled in accordance with the current data protection laws and ethical guidelines. Requests to access the datasets should be directed to s.chiou@bham.ac.uk.

## Ethics Statement

The studies involving human participants were reviewed and approved by West Midlands—South Birmingham Research Ethics Committee. The patients/participants provided their written informed consent to participate in this study. Written informed consent was obtained from the individual(s) for the publication of any potentially identifiable images or data included in this article.

## Author Contributions

Both authors contributed to the study concept and design, data acquisition and analysis, drafting the manuscript and figures, and manuscript proofreading.

## Conflict of Interest

The authors declare that the research was conducted in the absence of any commercial or financial relationships that could be construed as a potential conflict of interest.
